# Hybrid Green Materials Obtained by PCL Melt Blending with Diatomaceous Earth

**DOI:** 10.3390/molecules29061203

**Published:** 2024-03-08

**Authors:** Maria Rosalia Carotenuto, Giuseppe Cavallaro, Ileana Chinnici, Giuseppe Lazzara, Stefana Milioto

**Affiliations:** 1 Dipartimento di Fisica e Chimica “E. Segrè”, Università degli Studi di Palermo, Viale delle Scienze, Pad. 17, 90128 Palermo, Italy; mariarosalia.carotenuto@unipa.it (M.R.C.); giuseppe.lazzara@unipa.it (G.L.); stefana.milioto@unipa.it (S.M.); 2 INAF—Astronomical Observatory “G. S. Vaiana”, Piazza del Parlamento, 1, 90134 Palermo, Italy; ileana.chinnici@inaf.it

**Keywords:** diatomaceous earth, composites, polycaprolactone, melt blending

## Abstract

In this work, diatomaceous earth (Diat) was explored as filler for polycaprolactone (PCL) to obtain composite green materials with promising viscoelastic and thermal properties. The composites were prepared by blending variable Diat amounts (5, 15 and 50 wt%) with a molten PCL matrix. The viscoelastic characteristics of PCL/Diat hybrids were studied by Dynamic Mechanical Analysis (DMA) under an oscillatory regime, while the thermal properties were determined by Differential Scanning Calorimetry (DSC) and Thermogravimetric Analysis (TGA). We detected that the presence of Diat enhances the energy storage capacity of PCL for temperatures lower than the polymer melting point. Both DMA and DSC data revealed that the PCL melting temperature is slightly affected by the Diat addition, while the TGA results showed that the thermal stability of the polymer can be significantly improved by mixing PCL with diatomaceous earth. Moreover, we observed that the dispersion of Diat into the matrix favors the crystallization process of PCL. Interestingly, the improvements of PCL properties (elasticity, thermal stability, and crystallinity) are proportional to the Diat concentration of the composites. These findings reflect the interfacial compatibility between PCL and diatomaceous earth. In conclusion, this study highlights that the preparation of PCL/Diat hybrids by melt blending is suitable for the development of composite materials for technological applications, including the remediation of air pollutants within museum environments.

## 1. Introduction

Diatomaceous earth (Diat) is a natural material derived by amorphous silica cell walls of dead diatoms deposited on marine sediments [1]. Chemically, Diat is composed of silicon dioxide (>90%), alumina and small quantities of iron oxides [2]. Moreover, the diatomaceous surface can contain impurities, including clays and calcium carbonate [3]. Diatomaceous earth possesses a large porosity (ca. 60%) with pores with a diameter ranging between 200 and 460 nm [4]. Diat particles are quite polydisperse in size and typically measure between 3 and 200 µm [5]. Diatomaceous earth can be used for numerous applications, including as a carrier for controlled release of functional drugs [6,7,8], adsorbent material for remediation [9,10] and reinforcement filler for polymers [11,12,13]. The literature reports that the mechanical properties of several polymers (polylactic acid [11], polyetherimide [12] and polyamide [13]) can be improved by dispersing Diat particles within the polymeric matrices. Diatomaceous earth is suitable for various environmental purposes. For example, Diat particles can be exploited as indicators of the quality of aquatic systems, as reported for the Zarafshan River in Uzbekistan [14]. Diat was successfully explored for the removal of atrazine and organophosphorus pesticides (parathion-methyl, chlorpyriphos, fenamiphos and methidathion) from river and waste waters [15]. Pornaroonthama et al. [16] proved that diatomaceous earth modified with a cationic surfactant (cetyltrimethylammonium bromide) and tetraethylenepentamine (TEPA) is effective in CO_2_ adsorption. The incorporation of carboxylic and amine functional groups on the surface of diatomaceous earth was explored to obtain adsorbent materials for zinc retention [17]. In this work, we investigated the suitability of diatomaceous earth as a filler for polycaprolactone (PCL), which is a biopolymer largely employed for biomedical and packaging purposes [18,19,20,21]. PCL is a biocompatible polymer suitable for numerous environmental applications, such as water remediation and air purification. It is reported that the combination of PCL with other biopolymers (chitosan and gelatin) leads to the fabrication of electrospun membranes with a removal capacity towards methylene blue from the aqueous phase [22]. PCL modified with thiol groups exhibited a high adsorption capacity towards Pb(II) and Cd(II) from water [23]. Filter membranes for wastewater treatment were fabricated by filling a PCL matrix with an amine-functionalized metal–organic framework [24]. In addition, elecrospun membranes based on PCL were used for air purification by the adsorption of both outdoor an indoor gaseous pollutants and bioaerosols [25]. Rao et al. evidenced that an air filter formed by PCL nanofibers is effective for capturing PM2.5 particles [26]. It was proved that PCL is effective as an anticorrosive coating of Mg alloys [27]. In the last decades, the addition of natural fillers (nanoclays, diatomaceous earth, nanocellulose, microwax, plants and animal fibers) within biopolymeric matrices was successfully exploited to improve the properties and functionalities of polymers, allowing one to extend their application areas [28,29,30,31,32,33,34]. The effects of diatomaceous earth on the PCL’s enzymatic degradation were investigated by Mitomo et al. [35]. Recently, PCL-bound diatomite filters for the removal of metal ions and micro/nanoplastics from water were fabricated by acetone mixtures [36]. It was detected that PCL/Diat composites possess a removal efficiency of ca. 90% towards cationic metal ions (Cu, Co, Pb, Cd, Cr). On the other hand, the adsorption capacity towards micro/nanoplastics depends on their specific sizes. The removal efficiency towards 1.1 µm plastics is up to 94%, while the adsorption capacity is slower (up to ca. 73%) for 100 nm plastics. The properties of biocomposite materials are strictly correlated with the adhesion between the polymer and filler [11,37,38]. The specific matrix/filler interactions can be studied by using different techniques, including thermal analysis methods. In this regard, the high interfacial interactions between PLA and Diat particles was evidenced by the enhancement of the polymer crystallinity detected by Differential Scanning Calorimetry (DSC) measurements [11]. Adhesion forces with the fillers can alter the polymer glass transition, which can be investigated by both DSC and Dynamic Mechanical Analysis (DMA) [32,39]. For instance, the presence of clay nanotubes within Funori induced a decrease of the glass transition temperature [32]. The addition of halloysite nanotubes reduced the heat capacity change related to the glass transition of Rosin 100, which is an esterified colophony [39]. As reported in the literature [40,41], an improvement of the polymer thermal stability can be attributed to barrier effect phenomena, which occur for composites with a homogeneous dispersion of the filler within the matrix. Namely, the presence of uniformly distributed fillers hinders the propagation of the volatile products generated by the polymer decomposition, inducing an increase in the thermal resistance to degradation. Thermal stabilization effects can be studied by Thermogravimetric Analysis (TGA), which also provides information on the organic/inorganic composition of biocomposite materials. Moreover, TGA is suitable for estimating the amount of water molecules physically adsorbed to both inorganic and organic materials [42,43,44]. Therefore, thermogravimetry can be explored to evaluate the influence of inorganic fillers on the moisture content of polymeric matrices. This work represents a starting step for the fabrication of PCL/Diat composites useful for environmental applications, since both components are biocompatible materials with relevant adsorption capacities towards inorganic and organic pollutants. In particular, further studies will be conducted to investigate the efficacy of PCL/Diat hybrids for air purification in museum showcases, which present high levels of volatile organic compounds (VOCs). For example, high concentrations of acetic and formic acid vapors are produced by the decomposition of wooden storage cabinets [45]. Recently, composite materials based on polymers and inorganic fillers were studied as adsorbents to improve the air quality in museum environments [46,47]. In this regard, we aim to fabricate biocompatible materials for the conservation of artworks.

## 2. Results and Discussion

### 2.1. Viscoelastic Properties of PCL/Diat Composites

**Figure 1:**
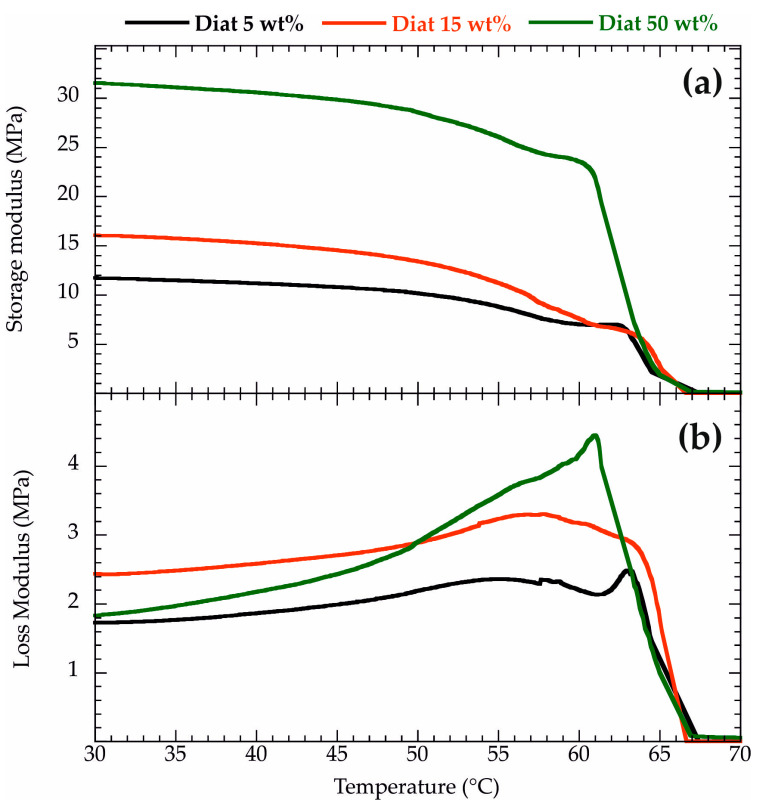
Storage (**a**) and loss (**b**) moduli as functions of temperature for PCL/Diat nanocomposites at variable compositions.

The viscoelastic properties of PCL/Diat composites were studied by Dynamic Mechanical Analysis (DMA) within an oscillatory regime. Specifically, we determined the rheological moduli at variable temperatures by performing DMA tests under a heating ramp. Figure 1 shows the storage (G′) and loss (G″) moduli as functions of the temperature for PCL filled with variable amounts of Diat.

As a general result, G′ exhibited a sharp decrease in the temperature range between 50 and 70 °C (Figure 1a) that reflects a worsening of the elastic capacity of the materials. Namely, the composites reduced their energy storage ability due to the PCL melting. According to this consideration, the G′ vs. Temperature trend of pure PCL (see Supplementary Materials) evidenced a similar trend. We calculated the temperature at the G′ inflection point (T_G′inf_) in the range of 50–70 °C for both pure PCL and PCL/Diat composites. As shown in Table 1, T_G′inf_ is slightly affected by the Diat addition within the PCL matrix, highlighting that the melting temperature is similar for pure polymer and composites. In general, G″ presents a maximum corresponding to T_G′inf_ (Figure 1b), evidencing that the materials possess the highest capacity to dissipate energy due to their viscous behavior.

**Figure 2:**
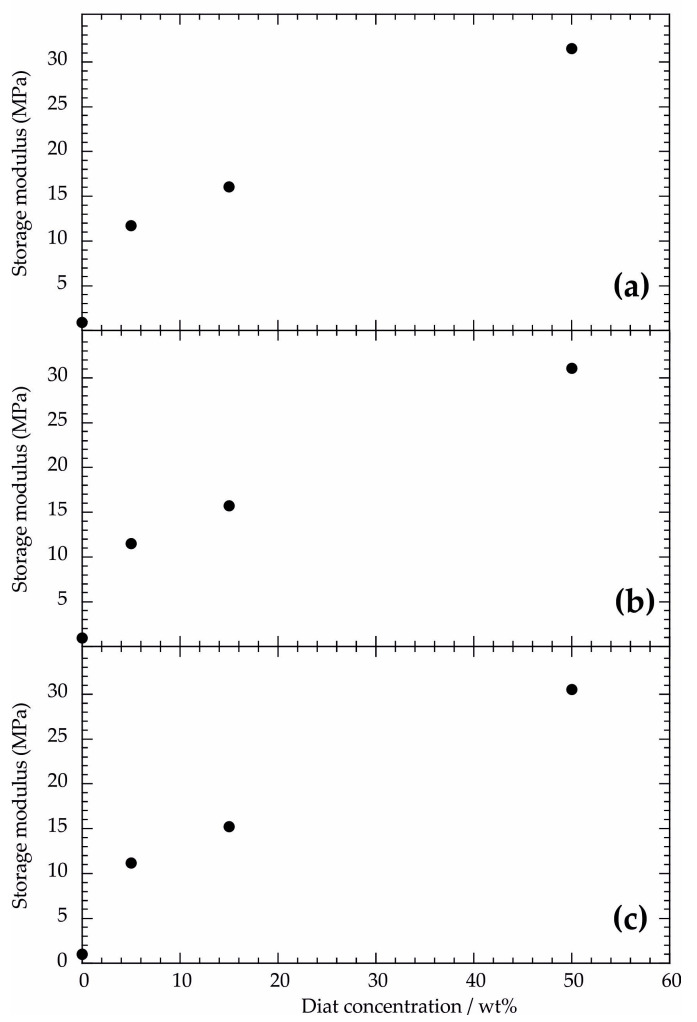
Storage modulus as a function of Diat concentration at 30 °C (**a**); 35 °C (**b**); and 40 °C (**c**).

We evaluated the viscoelastic properties of the composites before PCL melting by considering the rheological moduli at temperatures lower than 50 °C. As evidenced in Figure 1, G′ shows a slight decreasing trend at 30–50 °C, ruling out any structural rearrangements of PCL/Diat composites within the mentioned temperature range. This finding was observed for pure PCL (see Supplementary Materials). Figure 2 shows the influence of the Diat concentration on the storage modulus at variable temperatures (30, 35 and 40 °C). We detected that G′ is enhanced by the PCL filling with Diat. Namely, the presence of the filler improved the PCL elasticity and, consequently, the storage energy ability during oscillating mechanical tests. These improvements are proportional to the Diat concentration. The enhancement of the storage energy capacity in the composite materials could be attributed to the adhesion of PCL on the Diat surface, in agreement with their good compatibility.

**Figure 3:**
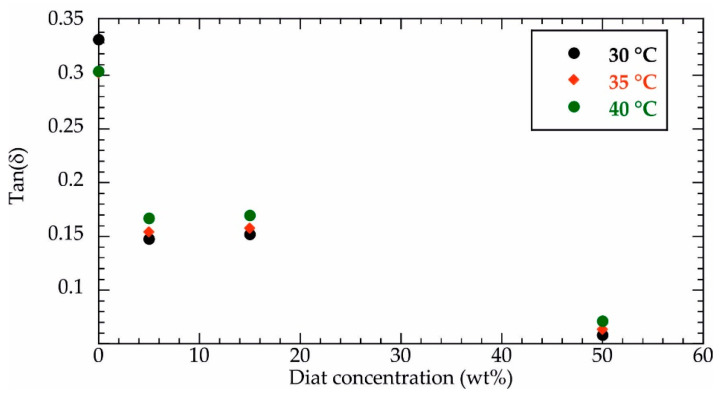
Tan(δ) as a function of Diat concentration at variable temperatures (30, 35 and 40 °C).

In addition to the storage modulus, we determined tan(δ) values of the composites at variable Diat filling amounts. It should be noted that tan(δ) was calculated as the G″/G′ ratio. As displayed in Figure 3, tan(δ) vs. Diat concentration shows decreasing trends for all investigated temperatures (30, 35 and 40 °C). Accordingly, we can state that the elastic contribution is enhanced by Diat addition within the PCL matrix, in agreement with the results reported in Figure 2. The PCL filling with small Diat amounts (5 and 15 wt%) generated tan(δ) reductions of ca. 50%, while tan(δ) variations were significantly larger (ca. 80%) for a Diat concentration of 50 wt%.

### 2.2. Effects of Diat Addition on the Melting and Crystallization Processes of PCL

**Figure 4:**
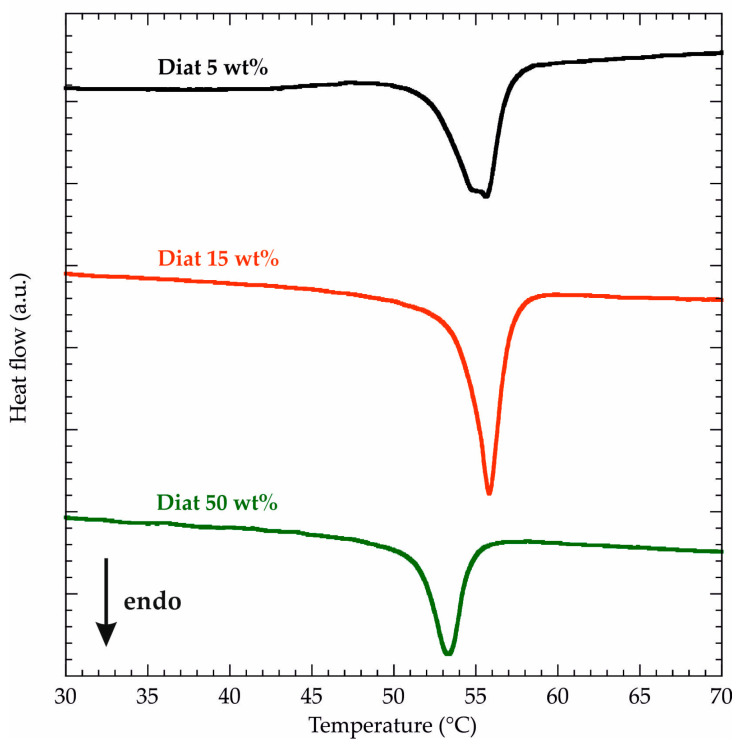
DSC curves (heating ramp) for PCL/Diat composites at variable compositions.

Differential Scanning Calorimetry (DSC) was used to investigate the influence of Diat addition on the melting and crystallization processes of PCL. Specifically, DSC experiments under a heating ramp allowed us to investigate the thermodynamics of PCL melting, while DSC measurements under a cooling ramp were helpful to determine the thermodynamic parameters of PCL crystallization. As shown in Figure 4, DSC curves obtained by heating PCL/Diat composites present an endothermic peak in the range of 50–60 °C that can be ascribed to polymer melting. Accordingly, the DSC curve of pure PCL (see Supplementary Materials) evidenced an endothermic signal within the same temperature range. We calculated the melting temperature (T_m_) values from the minima of the DSC endothermic signals, while the corresponding enthalpy variations (ΔH_m_) were estimated from the integration of the peaks. The obtained data are collected in Table 2.

**Figure 5:**
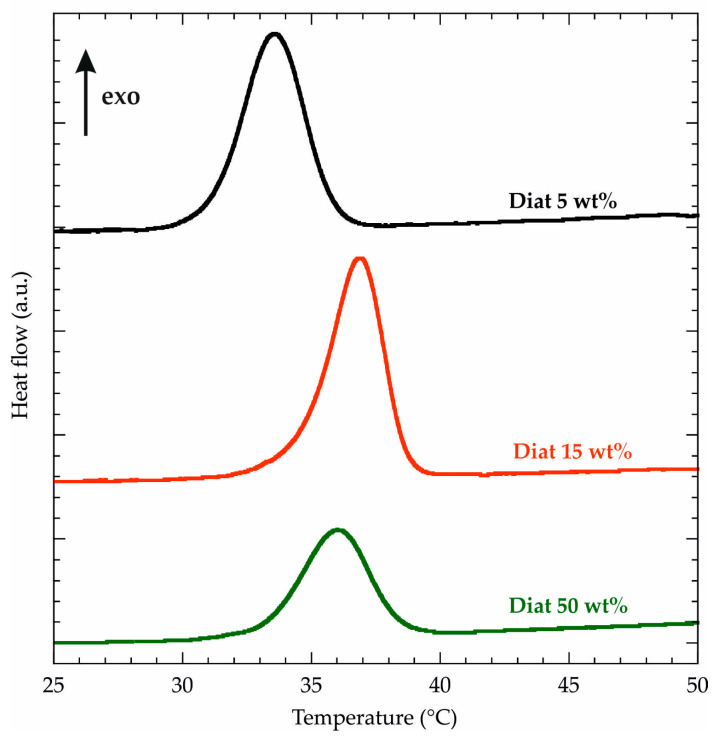
DSC curves (cooling ramp) for PCL/Diat composites at variable compositions.

We detected that T_m_ is slightly reduced (up to 4 °C) after the Diat addition within the PCL matrix. On the other hand, PCL/Diat composites exhibited larger ΔH_m_ values compared to pure PCL. We determined that the presence of 50 wt% Diat generates an increase of ca. 14% of PCL melting enthalpy. As reported in the literature [48,49], the presence of fillers within the polymeric matrix can produce a ΔH_m_ decrease, indicating the worsening of the polymer crystallinity. Opposite results (ΔH_m_ increase) indicate that the fillers act as nucleating sites for the polymer crystallization [11]. Accordingly, we can hypothesize that diatomaceous earth dispersed in the polymeric matrix favors the crystal nucleation of PCL chains. Within this, Zglobicka et al. [11] evidenced that Diat particles act as nucleating agents for polylactic acid (PLA), increasing the polymer crystallinity. These results confirm the compatibility between PCL and Diat in the composites. Similar findings were detected for PCL/clays [50] and polylactide/layered silicates [51]. Figure 5 shows the DSC curves of PCL/Diat composites obtained by a cooling ramp. As expected, we observed an exothermic signal for all the composite materials due to the PCL crystallization. We determined the crystallization temperature (T_c_) by the maxima of the peaks, while the crystallization enthalpy variation (ΔH_c_) was calculated by integrating the exothermic signals. The thermodynamic parameters of the PCL crystallization are summarized in Table 3, which also reports the data obtained from the analysis of the DSC curve under cooling for pure PCL (see Supplementary Materials).

We detected that T_c_ is not altered by the presence of diatomaceous earth, except for PCL/Diat 5 wt%, which evidenced a slight reduction (ca. 3 °C) of the crystallization temperature. For the crystallization enthalpy, we observed larger ΔH_c_ absolute values for PCL/Diat composites than for pure polymer. These results confirm that the filler addition favors the crystallization process of PCL. The highest ΔH_c_ enhancement (ca. 12%) was estimated for PCL/Diat 50 wt%. It should be noted that the T_m_ decrease (Table 2) in the composites is caused by the degree of crystal perfection, which can be found in the enthalpy of crystallization.

### 2.3. Thermal Stability of PCL/Diat Composites

**Figure 6:**
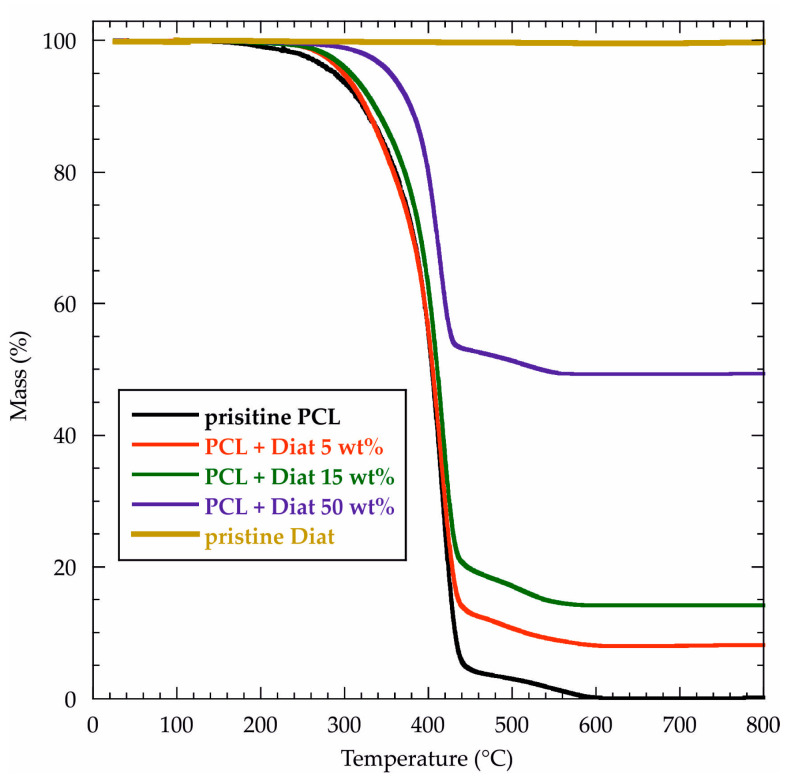
TG curves for pristine PCL, pristine Diat and PCL/Diat composites at variable compositions.

The thermal stability of PCL/Diat composites was investigated by Thermogravimetric Analysis (TGA). Figure 6 compares the thermogravimetric (TG) curves of the composites with those of their pure components (PCL and Diat).

We observed that diatomaceous earth (inorganic filler) was thermally stable up to 800 °C, while PCL (organic polymer) was completely degraded at 600 °C. As expected, the residual matters at 800 °C (MR_800_) of PCL/Diat composites range between 0 and 100 wt% because they present both inorganic and organic molecules in their composition. The literature reports similar results for polymer/nanoclay hybrids [52]. Table 4 collects the MR_800_ values for all composite materials. Clearly, we estimated that MR_800_ is proportional to the Diat content of the composites.

The TG curves (Figure 6) evidenced that both pure PCL and PCL/Diat composites present two mass losses within ca. 200–600 °C that represent the temperature range of polymer decomposition. We evaluated the thermal stability of the materials from the temperature at the onset point of the first mass change, which is predominant for all the samples. As evidenced in Figure 3, the Diat addition within the PCL matrix induced significant increases of the onset temperature (T_ons_), highlighting that the composites are thermally more stable compared to pure polymer. Specifically, T_ons_ was enhanced by 11, 19 and 41 °C for Diat concentrations of 5, 15 and 50 wt%, respectively. In general, the improvement of the thermal stability for composites with a homogeneous morphology might be attributed to the barrier effect of the inorganic fillers towards the volatile products generated from the polymer degradation [52]. This consideration is appropriate when interpreting the TGA results of PCL/Diat composites. The homogeneity of the composite materials highlights that PCL and Diat are interfacially compatible. Furthermore, the TG curves (Figure 6) evidenced that the PCL and PCL/Diat composites are hydrophobic, since they do not present any mass loss at 25–150 °C, which is the temperature range for the evaporation of water molecules physically adsorbed onto the surface of inorganic/organic materials.

## 3. Materials and Methods

### 3.1. Chemicals

Diatomaceous earth (Diat) and polycaprolactone (PCL) are from Sigma Aldrich (St. Louis, MO, USA). Both products were used without any purification. The chemical formulas of Diat and PCL are SiO_2_ and (C_6_H_10_O_2_)_n_, respectively. The average molecular mass of PCL is 80,000 g mol^−1^.

### 3.2. Preparation of PCL/Diat Composites

The composites were prepared through a melt blending procedure [53] by physically mixing Diat powder with molten PCL. In detail, 5 g of PCL were poured within a ceramic mortar, which was heated at 80 °C to guarantee that the polymer was in a molten state. Then, variable amounts of Diat powder were added within the mortar kept at 80 °C. Finally, molten PCL and Diat powders were mixed with a ceramic pestle for 15 min. The obtained materials were kept in a desiccator at 25 °C prior to their characterization. Table 5 collects the masses of PCL and diatomaceous earth employed for the preparation of the composites with variable compositions.

### 3.3. Characterization of PCL/Diat Composites

#### 3.3.1. Dynamic Mechanical Analysis (DMA)

DMA experiments were conducted by means of DMA Q800 (TA Instruments, Milan, Italy), using a shear sandwich clamp. The measurements were carried out in an oscillatory regime (frequency of 1 Hz and strain amplitude of 0.5%) under a heating ramp, allowing one to determine the influence of the temperature on the viscoelastic properties of the PCL/Diat composites. The heating ramp was set at 2 °C min^−1^ within an interval of 30 and 70 °C. The surface of the investigated samples was 1 cm^2^, while their thickness ranged between 0.4 and 1.4 mm.

#### 3.3.2. Differential Scanning Calorimetry (DSC)

DSC measurements were performed using micro-DSC III 106 (Setaram, Lyon, France) under Nitrogen atmosphere. Heating and cooling ramps were conducted within a range of 25 to 80 °C to investigate PCL melting and crystallization, respectively. The scanning rate was set at 1 °C min^−1^, while the mass of each sample was ca. 5 mg. It is important to evidence that the samples were subjected to two heating/cooling cycles in order to investigate the thermal history of PCL/Diat composites. In detail, the following thermal procedure was used: isotherm at 25 °C for 1 min, ramp 1 °C min^−1^ from 25 to 80 °C, isotherm at 80 °C for 1 min, and ramp 1 °C min^−1^ from 80 to 25 °C. This cycle was repeated two times. DSC curves (Figure 4 and Figure 5 and Supplementary Materials) and the corresponding thermodynamic parameters (Table 2 and Table 3) of melting and crystallization processes refer to the second cycle of measurements.

#### 3.3.3. Thermogravimetric Analysis (TGA)

TGA tests were carried out through Q5000 IR (TA Instruments, Milan, Italy) under Nitrogen atmosphere. The gas flows were set at 25 and 10 cm^3^ min^−1^ for the sample and the balance, respectively. The samples (ca. 5 mg) were heated from 25 to 800 °C using a scanning rate of 20 °C min^−1^. According to the literature [54], the temperature calibration of the apparatus was conducted on the basis of the Curie temperatures of standards (nickel, cobalt and their alloys).

## 4. Conclusions

Green composites based on biocompatible components (polycaprolactone and diatomaceous earth) were successfully prepared by melt blending procedure. The concentration of diatomaceous earth was systematically varied from 5 to 50 wt%. The elasticity of polycaprolactone at 30–40 °C (before the PCL melting point) was improved by dispersing Diat within the polymeric matrix. This effect is stronger for composites with a high Diat content. In this regard, we calculated tan(δ) reductions of ca. 50% for Diat concentrations of 5 and 15 wt%, whereas tan(δ) decreased by ca. 80% for a Diat content of 50 wt%. Based on the DSC results, we detected that Diat can act as a nucleating site for PCL crystallization. Accordingly, the absolute values of PCL melting and crystallization enthalpies were enhanced by the presence of diatomaceous earth in the matrix. On the other hand, the variations on the melting and crystallization temperatures were negligible. The mixing with Diat generated relevant effects on the PCL thermal stability. According to TGA data, we observed that PCL can be thermally stabilized by dispersing diatomaceous earth into the polymer matrix due to the barrier effect of the filler. The highest thermal stabilization (ca. 41 °C) was calculated for the PCL/Diat 50 wt% composite. As a general consideration, the results obtained by thermal analysis methods highlighted the interfacial compatibility between PCL and diatomaceous earth. In conclusion, this work demonstrated that melt blending of PCL with diatomaceous earth represents an easy and efficient strategy to obtain biocompatible composites with viscoelastic and thermal properties that are improved when compared with pure polymer.

## Figures and Tables

**Table 1:** Temperature at G’ inflection point.

Sample	T_G′inf_ (°C)
PCL	66.0
PCL/Diat 5 wt%	65.2
PCL/Diat 15 wt%	65.3
PCL/Diat 50 wt%	65.6

**Table 2:** Thermodynamic parameters of PCL melting obtained by DSC curves (heating ramp).

Sample	T_m_ (°C)	ΔH_m_ (J g_PCL_^−1^)
PCL	57.3	68.0
PCL/Diat 5 wt%	55.6	69.2
PCL/Diat 15 wt%	55.8	76.6
PCL/Diat 50 wt%	53.3	78.1

**Table 3:** Thermodynamic parameters of PCL melting obtained by DSC curves (cooling ramp).

Sample	T_c_ (°C)	ΔH_c_ (J g_PCL_^−1^)
PCL	36.7	−68.4
PCL/Diat 5 wt%	33.6	−72.3
PCL/Diat 15 wt%	36.9	−72.1
PCL/Diat 50 wt%	36.0	−76.1

**Table 4:** Thermogravimetric parameters of pure PCL, pure Diat and PCL/Diat composites at variable compositions.

Sample	T_ons_ (°C)	MR_800_ (%)
PCL	311	0
PCL/Diat 5 wt%	322	8.07
PCL/Diat 15 wt%	330	14.1
PCL/Diat 50 wt%	362	49.3
Diat	/	99.5

**Table 5:** Amounts of PCL and Diat mixed for the preparation of the composites at variable compositions.

Sample	Mass PCL/g	Mass Diat/g
PCL	5.00	0
PCL/Diat 5 wt%	5.00	0.26
PCL/Diat 15 wt%	5.00	0.88
PCL/Diat 50 wt%	5.00	5.00

## Data Availability

The data presented in this study are available in article and Supplementary Materials.

## Supplementary Materials

The following supporting information can be downloaded at: https://www.mdpi.com/article/10.3390/molecules29061203/s1, Figure S1: Storage and loss moduli as a function of temperature for pure PCL; Figure S2: DSC curve (heating ramp) for pristine PCL; Figure S3: DSC curve (cooling ramp) for pristine PCL.
