# Supplementary material for: Hybrid Green Materials Obtained by PCL Melt Blending with Diatomaceous Earth
Source: Molecules. 2024 Mar 8;29(6):1203. doi: 10.3390/molecules29061203 (PMC10975038; doi:10.3390/molecules29061203)
Supplement: Supplementary file 1 [file molecules-29-01203-s001.zip › molecules-2903084-supplementary.pdf]

molecules

Article

Hybrid Green Materials Obtained by PCL Melt Blending with Diatomaceous Earth

Maria Rosalia Carotenuto [1,2], Giuseppe Cavallaro [1,*], Ileana Chinnici [2], Giuseppe Lazzara [1] and Stefana Milioto [1]

1 Dipartimento di Fisica e Chimica "E. Segrè", Università degli Studi di Palermo, Viale delle Scienze, Pad. 17, 90128 Palermo, Italy; mariarosalia.carotenuto@unipa.it (M.R.C.); giuseppe.lazzara@unipa.it (G.L.); stefana.milioto@unipa.it (S.M.)
2 INAF—Astronomical Observatory "G. S. Vaiana", Piazza del Parlamento, 1, 90134 Palermo, Italy; ileana.chinnici@inaf.it
* Correspondence: giuseppe.cavallaro@unipa.it

Supplementary Materials

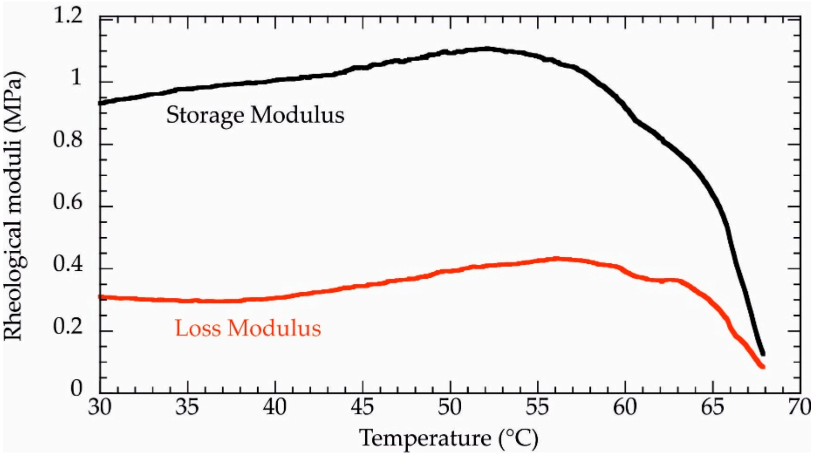

Figure S1. Storage and loss moduli as functions of temperature for pristine PCL.

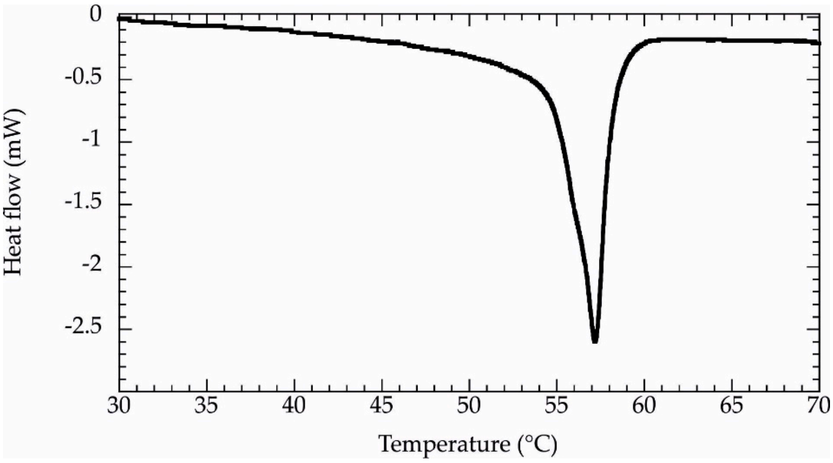

Figure S2. DSC curve (heating ramp) for pristine PCL.

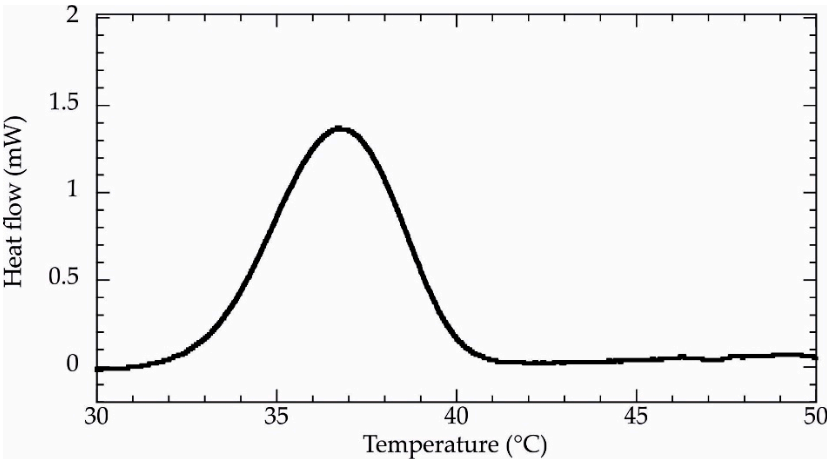

Figure S3. DSC curve (cooling ramp) for pristine PCL.
